# Imaging features of intrahepatic cholangiocarcinoma mimicking a liver abscess: an analysis of 8 cases

**DOI:** 10.1186/s12876-021-02002-1

**Published:** 2021-11-12

**Authors:** Manrong Liu, Jiong Chen, Ruisui Huang, Jianning Huang, Lin Li, Yunqian Li, Mi Qin, Wenqi Qin, Haiyang Nong, Ke Ding

**Affiliations:** 1 Department of Ultrasound, Guangxi Medical University Third Affiliated Hospital, Nanning, 530031 China; 2 Department of Radiology, Guangxi Medical University Third Affiliated Hospital, Nanning, 530031 China; 3 Department of Hepatobiliary Surgery, Guangxi Medical University Third Affiliated Hospital, Nanning, 530031 China; 4 Department of Pathology, Guilin Medical University First Affiliated Hospital, Guilin, 541001 China

**Keywords:** Intrahepatic cholangiocarcinoma, Liver abscess, Ultrasonography, Computed tomography, Magnetic resonance imaging

## Abstract

**Background:**

In rare cases, intrahepatic cholangiocarcinoma can present as a pyogenic liver abscess and are often misdiagnosed. This study aimed to analyze the imaging features of intrahepatic cholangiocarcinoma mimicking a pyogenic liver abscess.

**Methods:**

The clinical data and imaging results of eight patients with pathologically confirmed intrahepatic cholangiocarcinoma mimicking a liver abscess were retrospectively collected.

**Results:**

The mean age was 58 years with a range of 46–68 years. Fever and leukocytosis were present in six patients. All the eight lesions were a single mass. Air–liquid levels were present in two patients. Only one patient showed hepatic lobar atrophy and hepatic capsule retraction. The double target sign of liver abscess was not noticed in the CT/MRI images of all eight patients. The inner wall of the lesion was rough and irregular, with multiple dot/patchy and wall nodule enhancements. The abscess wall and the marginal parenchyma were supplied by the hepatic artery in four patients, and the intralesional arteries were rough and disrupted. Bile duct dilatation was seen adjacent to the lesion. In seven patients, diffusion-weighted images showed irregular patchy restricted diffusion in the marginal parenchyma of the necrotic area in addition to the prominent restricted diffusion in the necrotic area. Two patients with cholangiolithiasis showed patchy slight CT hypodensity, slight T1 hypointensity, slight T2 hyperintensity, and patchy delayed enhancement. Multiple lymph nodes enlargement in the hepatic hilar area and the retroperitoneal space were seen in five patients.

**Conclusion:**

Intrahepatic cholangiocarcinoma mimicking a pyogenic liver abscess have unique imaging features and require careful image examination to avoid misdiagnosis.

## Background

Intrahepatic cholangiocarcinoma on CT features slight hypodensity, an ill-defined rim, and delayed enhancement [1]. Ductal dilatation within or surrounding the lesion is seen with shrinkage of the affected lobe and hepatolithiasis. MRI and ultrasonography are also useful in diagnosing intrahepatic cholangiocarcinoma with similar features to that of CT [2–4]. In rare cases, intrahepatic cholangiocarcinoma can present as a pyogenic liver abscess and is often misdiagnosed [5, 6]. Considering the dismal prognosis of intrahepatic cholangiocarcinoma, misdiagnosing of this malignancy is devastating for the patient.

Radiologic study is the first choice for diagnosing pyogenic liver abscess. However, only a few case reports on radiologic features of intrahepatic cholangiocarcinoma mimicking abscess have been published [7, 8]. The present study aimed to review the radiological findings of 8 cases of intrahepatic cholangiocarcinoma presenting as pyogenic liver abscess and tried to identify the radiologic features that are useful in differentiate these two diseases.

## Methods

### Patients

All patients (n = 87) with pathologically confirmed intrahepatic cholangiocarcinoma who were treated at our hospital between 2012 and 2020 were identified. Among them, 8 patients presented to our hospital with pyogenic live abscess but were finally diagnosed with intrahepatic cholangiocarcinoma. The clinical data and radiologic findings of these 8 patients were retrospectively collected. This study was approved by the ethics committee of our hospital.

### Imaging methods

Three patients were examined using Doppler ultrasonography with a 3.0–5.0 MHz SC6-1 transducer (Aixplorer, SuperSonic Imagine, France).

All the 8 patients were examined by plain CT and enhanced CT (Discovery CT750HD, GE, USA). The CT parameters included 120 kV tube voltage, automatic tube current modulation, 0.625 mm × 64 detectors, 0.984:1 pitch, 512 × 512 matrix, 5 mm slice thickness, 5 mm slice interval, and 1.25 mm reconstruction. For enhanced scanning, 1.5 mL/kg Iopromide (300 mgI/ml) was injected via the antecubital vein at a rate of 3 mL/s. Images of the late arterial phase, the portal venous phase, and the equilibrium phase were obtained during 30–35 s, 55–60 s, and 120–125 s after the injection.

Seven out of the 8 patients received MRI examination (Verio, 3.0 T, Siemens, Germany) including T1-weighted imaging, T2-weighted imaging, diffusion-weighted imaging, and enhanced imaging. For enhanced MRI, 0.1 mmol/kg gadopentetate dimeglumine (Schering, Berlin, Germany) was injected at a rate of 3 mL/s. Images of the early arterial phase, the late arterial phase, the portal venous phase, the equilibrium phase, and the delayed phase were obtained at 10 s, 19 s, 25 s, 90 s, and 180 s after the injection.

### Image analysis

All medical images were analyzed by three senior radiologists. Consensus of the diagnosis was reached by discussion.

## Results

### Patient characteristics

The general clinical data of the 8 patients are show in Table 1. All patients were initially diagnosed with pyogenic liver abscess and were treat with antibiotics. However, the abscesses did not resolve. Further biopsy and surgical resection confirmed intrahepatic cholangiocarcinoma with necrosis and abscess. All the resected tumors were positive for carbohydrate antigen 19–9, cytokeratin 19, cytokeratin 7, and Ki-67. All the tumors were single lesions of the mass-forming type according to the classification of primary liver cancer proposed by the Liver Cancer Study Group of Japan [9].

**Table 1:** Demographic and clinical characteristics of the patients

Patient no	Sex	Age	Cirrhosis	HBsAg	Alpha fetoprotein	Fever	Leukocytosis	Diabetes
1	Male	57	−	−	−	−	−	−
2	Male	63	+	+	−	+	+	+
3	Male	58	−	−	−	+	+	+
4	Male	46	−	−	−	+	+	+
5	Male	68	−	−	−	+	+	+
6	Male	56	−	−	−	+	+	+
7	Female	62	−	−	−	+	+	+
8	Female	51	−	−	−	−	−	−

### Case presentation

**Fig. 1:**
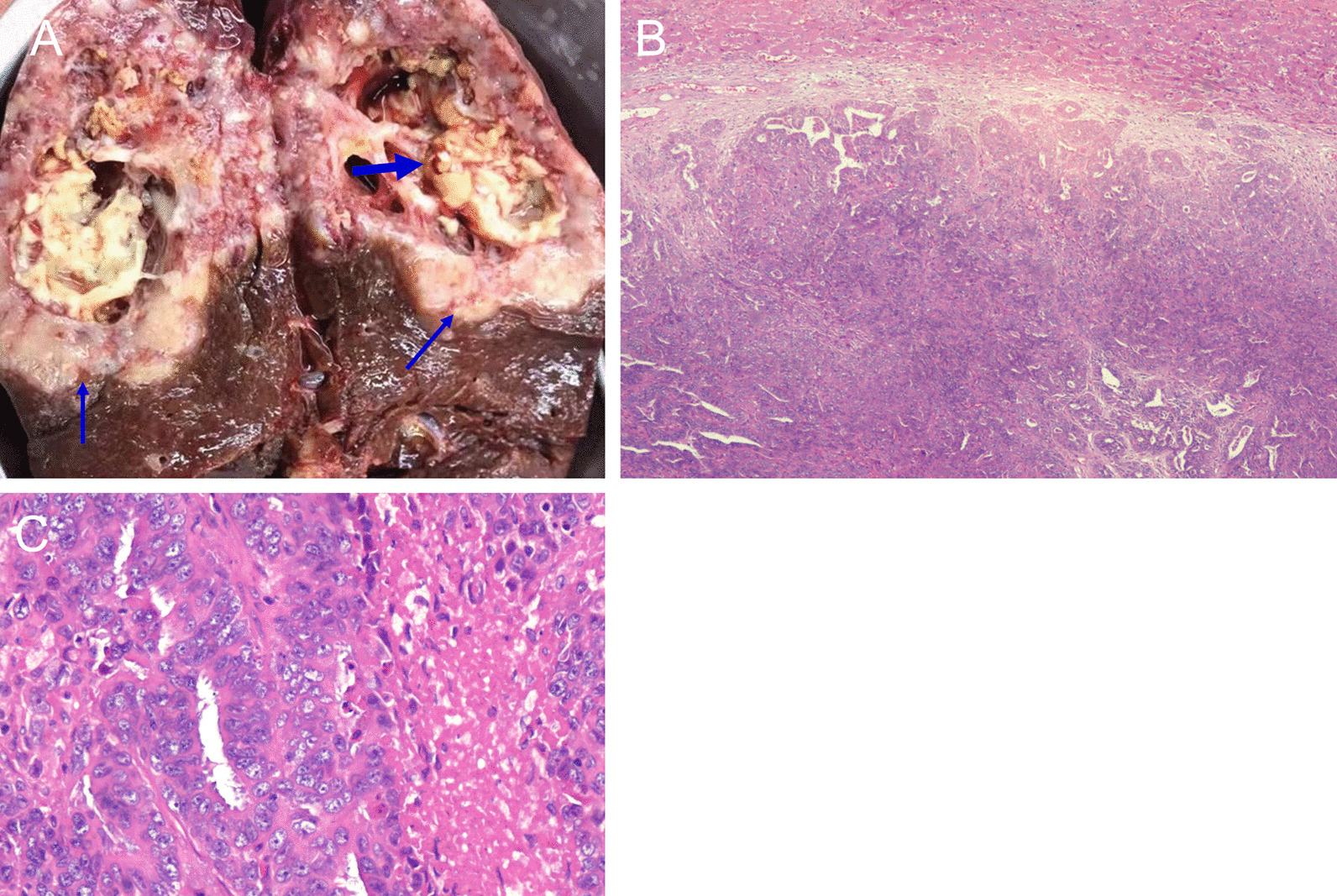
Pathological examination of the liver lesion of case 1. **A** Gross appearance at the cut surface of the resected liver lesion showed the tumor (thin arrows) and the necrotic tissue and abscess (thick arrows). Hematoxylin and eosin staining of the cancerous tissue (**B** 40×; **C** 200×)

*Case 1* A 58-year-old man was diagnosed with poorly differentiated intrahepatic cholangiocarcinoma accompanied by intratumoral abscess in the right hepatic lobe (Fig. 1). Immunostaining results were Hepato (−), CK8 (+++), CEA (+), p53 (+), CK19 (+++), CD34 (−), CK (+++), CK7 (+), KI67 (+, 70%), S-100 (−), EMA (+++), and CD68 (−).

**Fig. 2:**
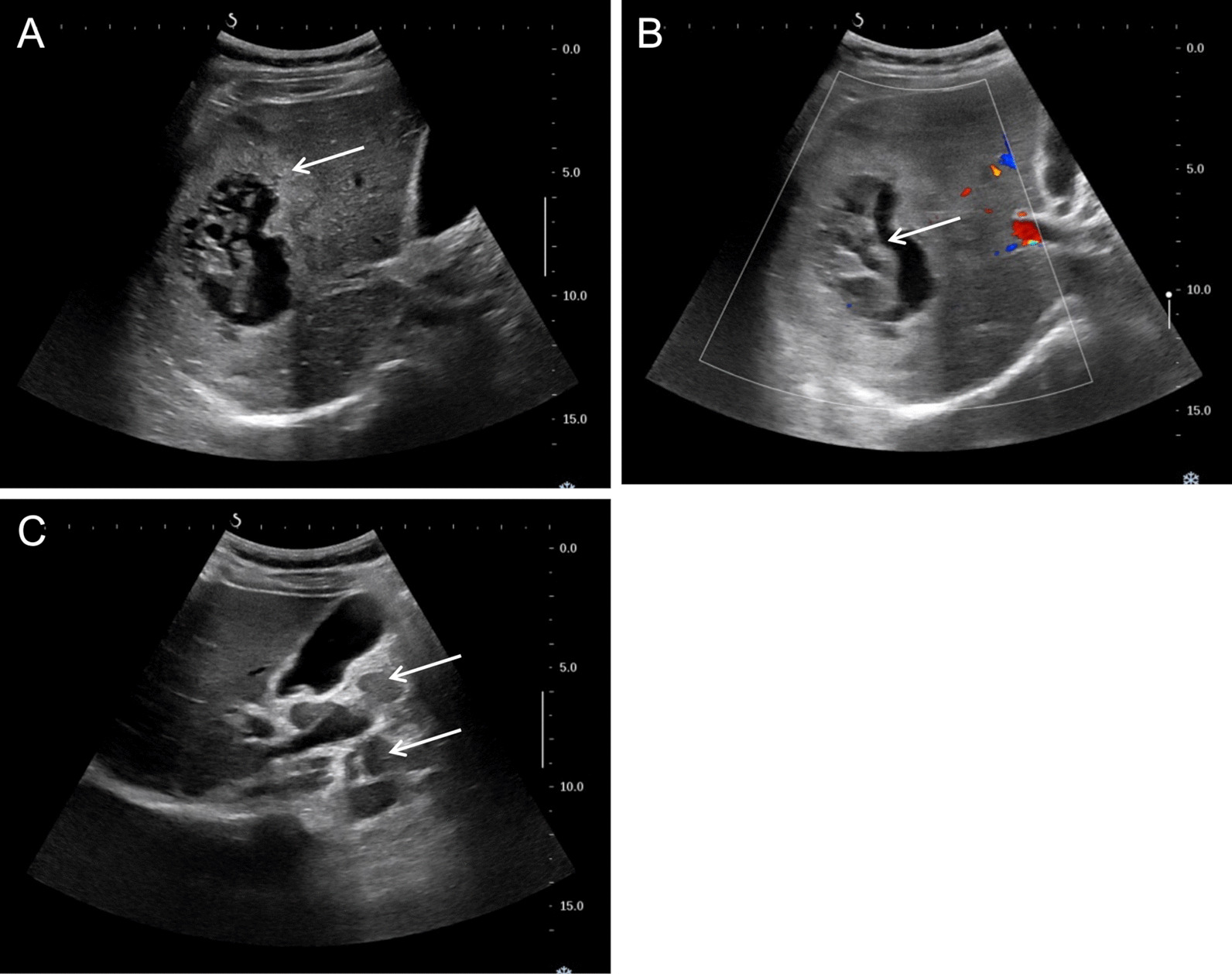
Ultrasonography images of case 1

Ultrasound showed a prominent edema belt surrounding the lesion (Fig. 2A, arrow) and an intralesional irregular area showing necrotic and liquidizing changes. Blood supply to the lesion was low (Fig. 2B, arrow). A hyperechoic belt was noticed posterior to the lesion, with multiple lymph nodes enlargement in the hepatic hilar area and the retroperitoneal space (Fig. 2C, arrows).

**Fig. 3:**
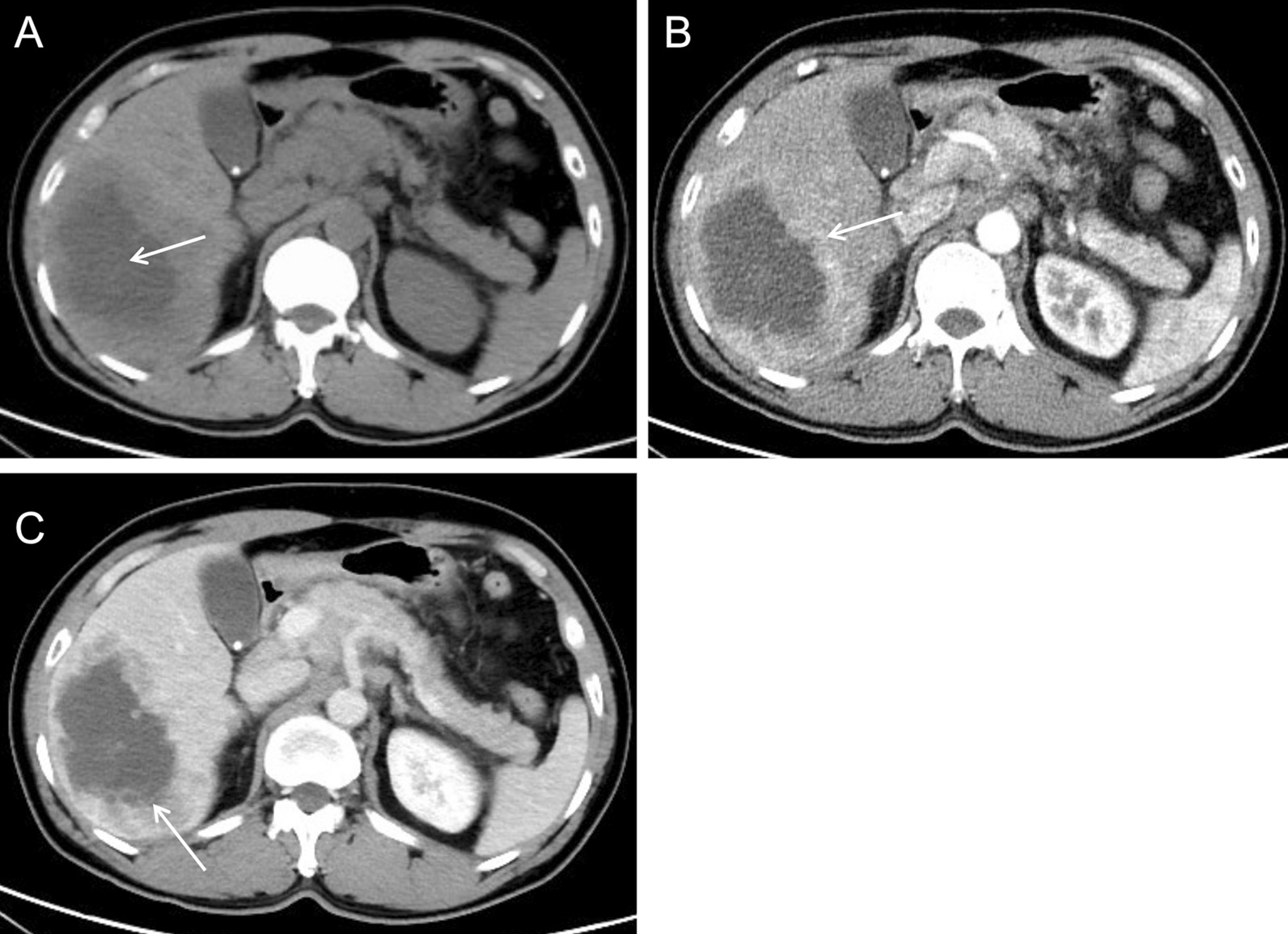
CT images of case 1

Plain CT showed a hypodense mass with an intralesional patchy area of hypodensity (Fig. 3A, arrow). Enhanced CT showed prominent enhancement of the lesion rim in the arterial phase (Fig. 3B, arrow) and decreased enhancement in the portal venous phase (Fig. 3C, arrow), which was a rapid-increase-rapid-decrease pattern. The double target sign of liver abscess was not noticed in the CT images. The abscess inner wall was rough and very irregular. Multiple dot/patchy and wall nodule enhancements were seen in the lesion. Multiple lymph nodes enlargement in the hepatic hilar area and the retroperitoneal space were also seen by CT.

**Fig. 4:**
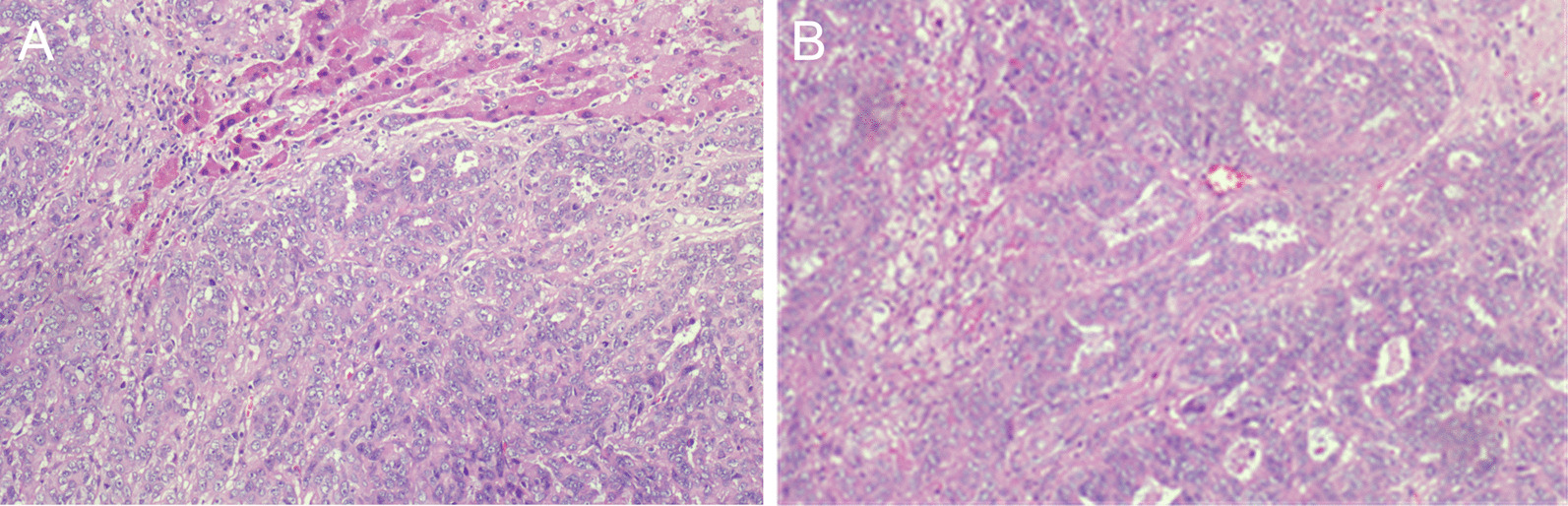
Hematoxylin and eosin staining of the cancerous tissue of case 2 showed poorly differentiated intrahepatic cholangiocarcinoma accompanied by necrosis and abscess (**A** 100×; **B** 200×)

*Case 2* A 56-year-old man was diagnosed with poorly differentiated intrahepatic cholangiocarcinoma accompanied by intratumoral abscess in the right hepatic lobe (Fig. 4). Immunostaining results were Hepato (−), CK8 (+++), CEA (+), P53 (+), CK19 (+++), CK7 (+), Ki67 (+ , 60%), and EMA (+++).

**Fig. 5:**
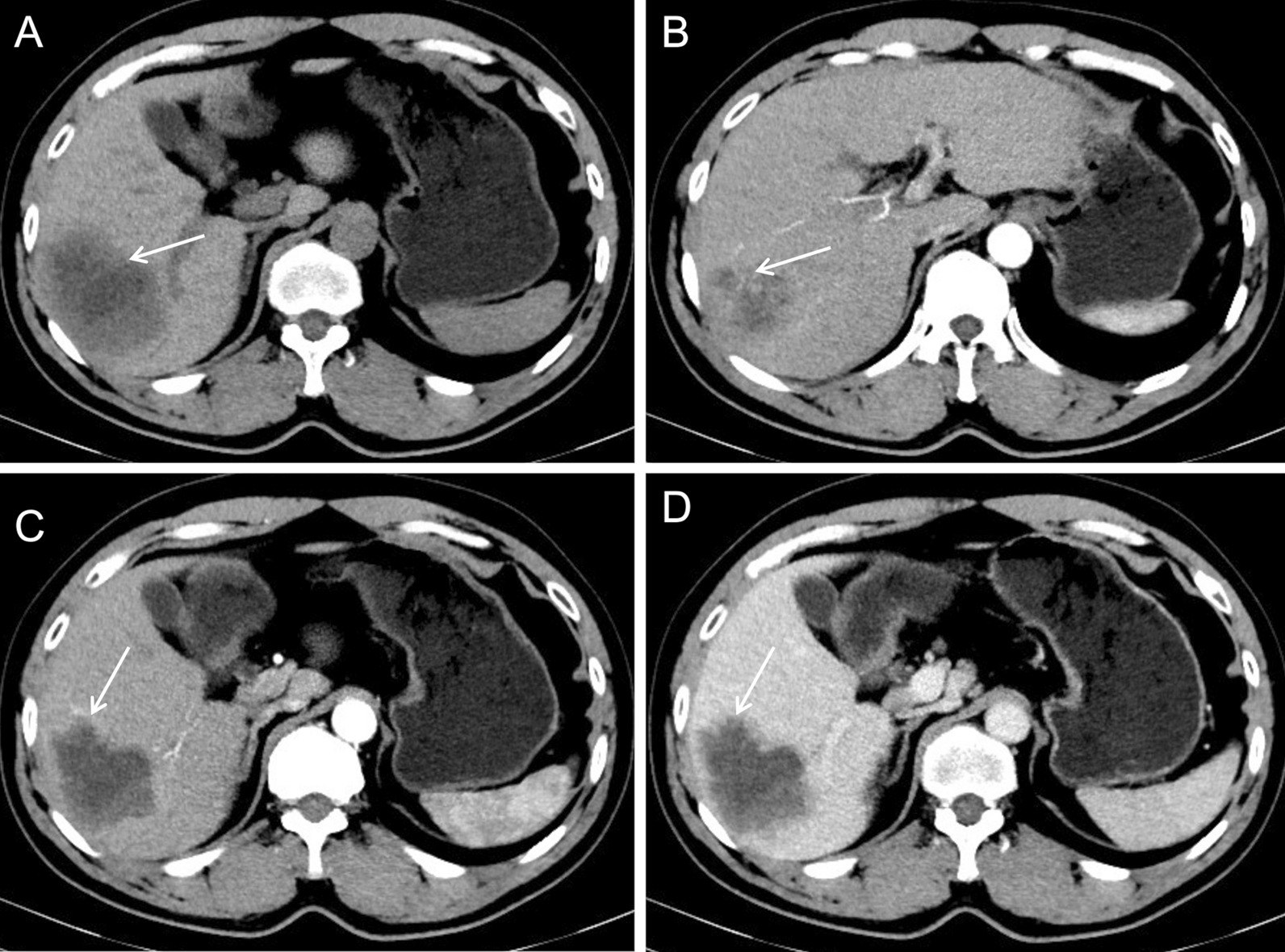
CT images of case 2

Plain CT showed a hypodense mass with an intralesional area of hypodensity showing necrotic and liquidizing changes (Fig. 5A, arrow). Arterial phase images of enhanced CT showed that the mass parenchyma was supplied by the hepatic artery, and that the intralesional arteries were rough and disrupted (Fig. 5B, arrow). The lesion rim showed irregular enhancement during the arterial phase (Fig. 5C, arrow), which was decreased during the venous phase. The double target sign of liver abscess was not noticed. The abscess inner wall was rough and irregular (Fig. 5D, arrow).

**Fig. 6:**
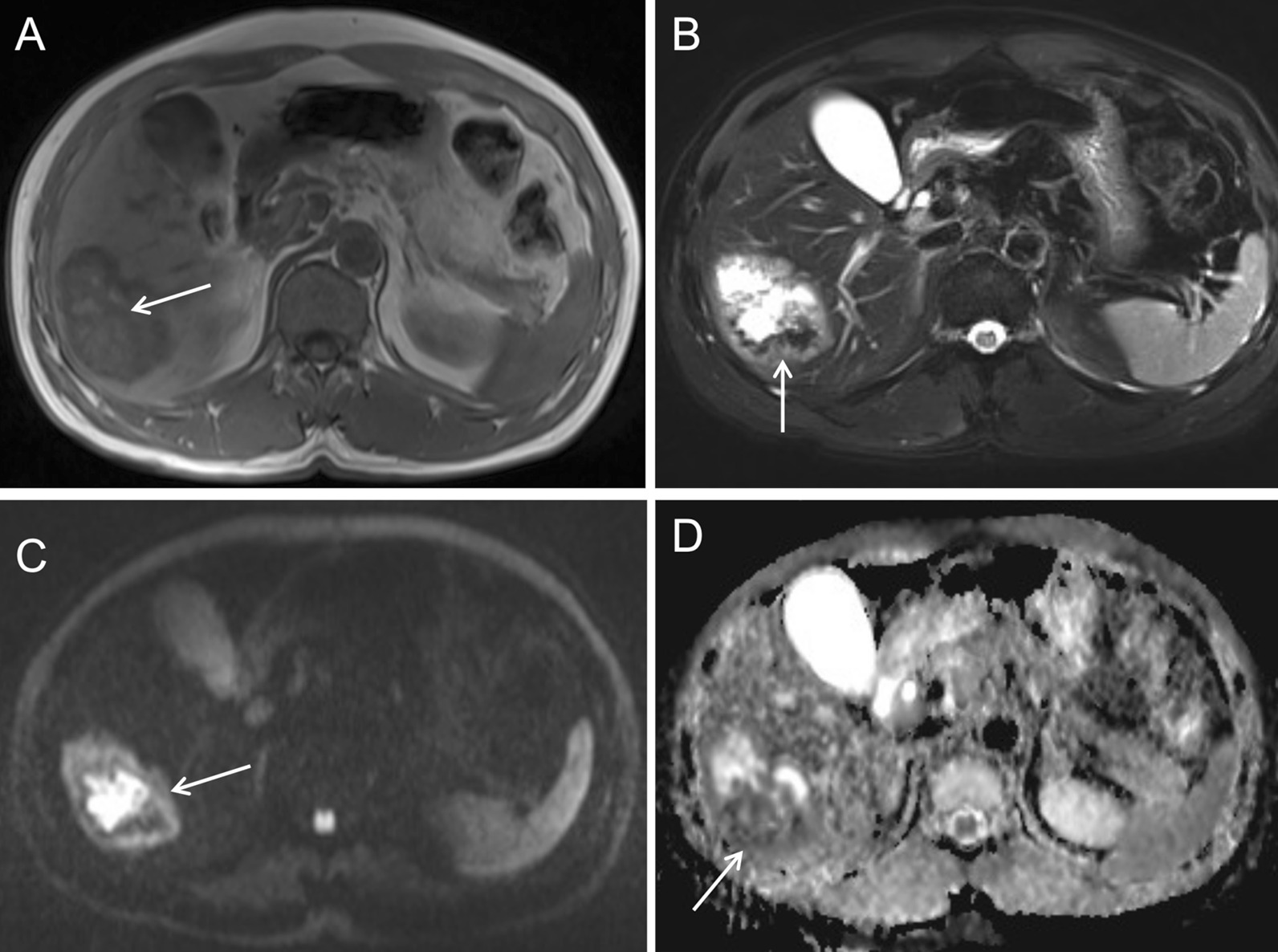
Plain MRI and diffusion-weighted images of case 2

T1-weighted images showed an uneven slight hypointense lesion, with multiple patchy hemorrhagic foci with slight hyperintensity (Fig. 6A, arrow). Fat-suppressed T2-weighted images showed irregular significant hyperintensity in the necrotic area of the lesion, and slight hyperintensity of the marginal parenchyma (Fig. 6B, arrow). Diffusion-weighted images and apparent diffusion coefficient images showed restricted diffusion of the necrotic area of the lesion and irregular ring-like restricted diffusion of the marginal parenchyma. Hyperintensity was found in the diffusion-weighted images and hypointensity in the apparent diffusion coefficient images (Fig. 6C, D, arrow).

**Fig. 7:**
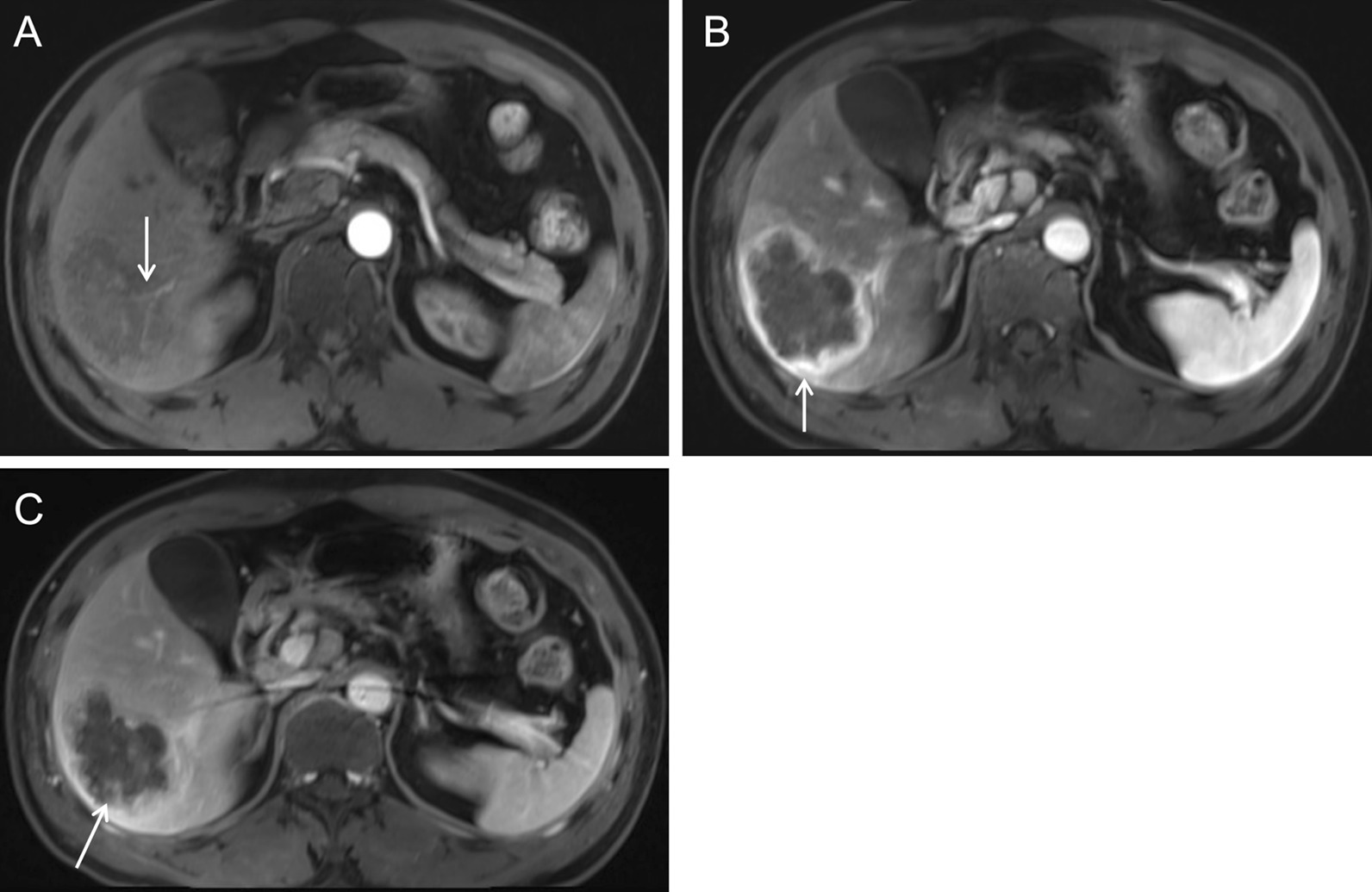
Enhanced MRI images of case 2

Arterial phase images of enhanced MRI showed that the lesion was supplied by the hepatic artery, and that the intralesional arteries were rough and disrupted (Fig. 7A, arrow). The lesion rim showed irregular ring-like significant enhancement during the arterial phase (Fig. 7B, arrow), which was decreased during the portal venous phase, showing a rapid-increase-rapid-decrease pattern. The double target sign of liver abscess was not noticed. The abscess inner wall was rough and very irregular. Multiple dot/patchy and wall nodule enhancements were seen in the mass (Fig. 7C, arrow).

**Fig. 8:**
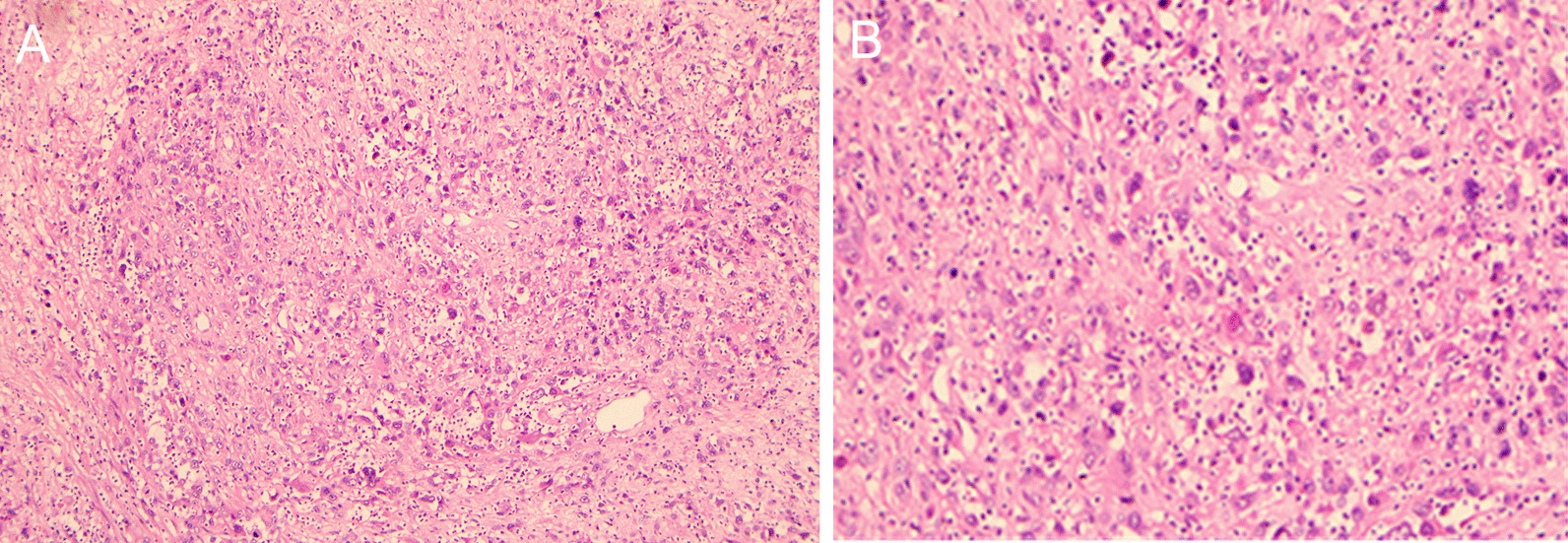
Hematoxylin and eosin staining of the cancerous tissue of case 2 showed poorly differentiated intrahepatic cholangiocarcinoma accompanied by necrosis and proliferation of granuloma (**A** 100×; **B** 200×)

*Case 3* A 68-year-man was diagnosed with poorly differentiated intrahepatic cholangiocarcinoma accompanied by intratumoral abscess in the left hepatic lobe (Fig. 8).

**Fig. 9:**
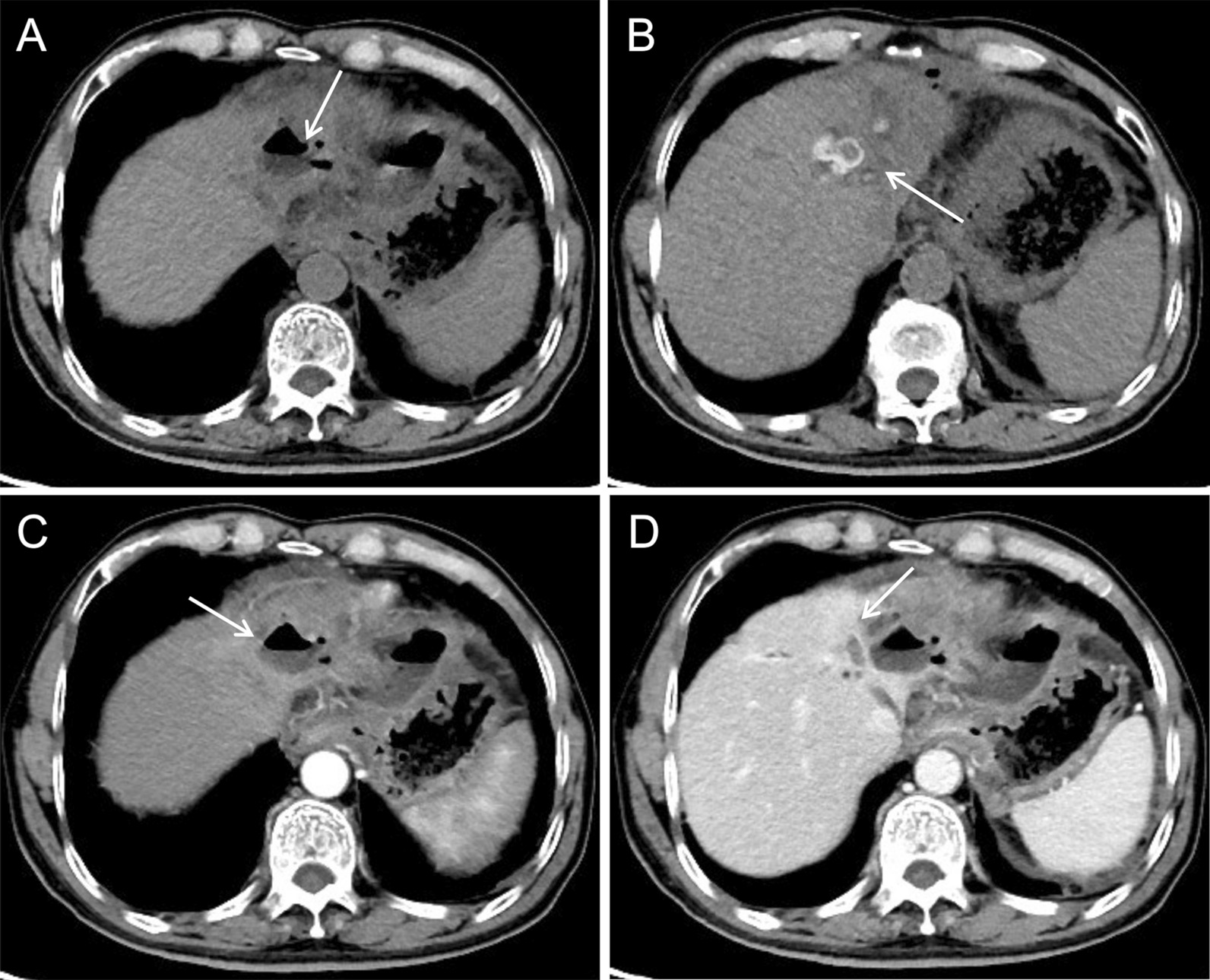
CT images of case 3

Plain CT showed an uneven hypodense mass with an intralesional area of hypodensity showing necrotic and liquidizing changes and an air-fluid level (Fig. 9A, arrow). The patient also had intrahepatic cholangiolithiasis and bile duct dilatation, which presented as a patchy slight hypodense opacity (Fig. 9B, arrow). Arterial phase images of enhanced CT showed that the lesion was supplied by the hepatic artery with slight enhancement (Fig. 9C, arrow). Portal venous images showed marginally blurred, patchy, delayed enhancement around the dilated bile duct (Fig. 9D, arrow).

**Fig. 10:**
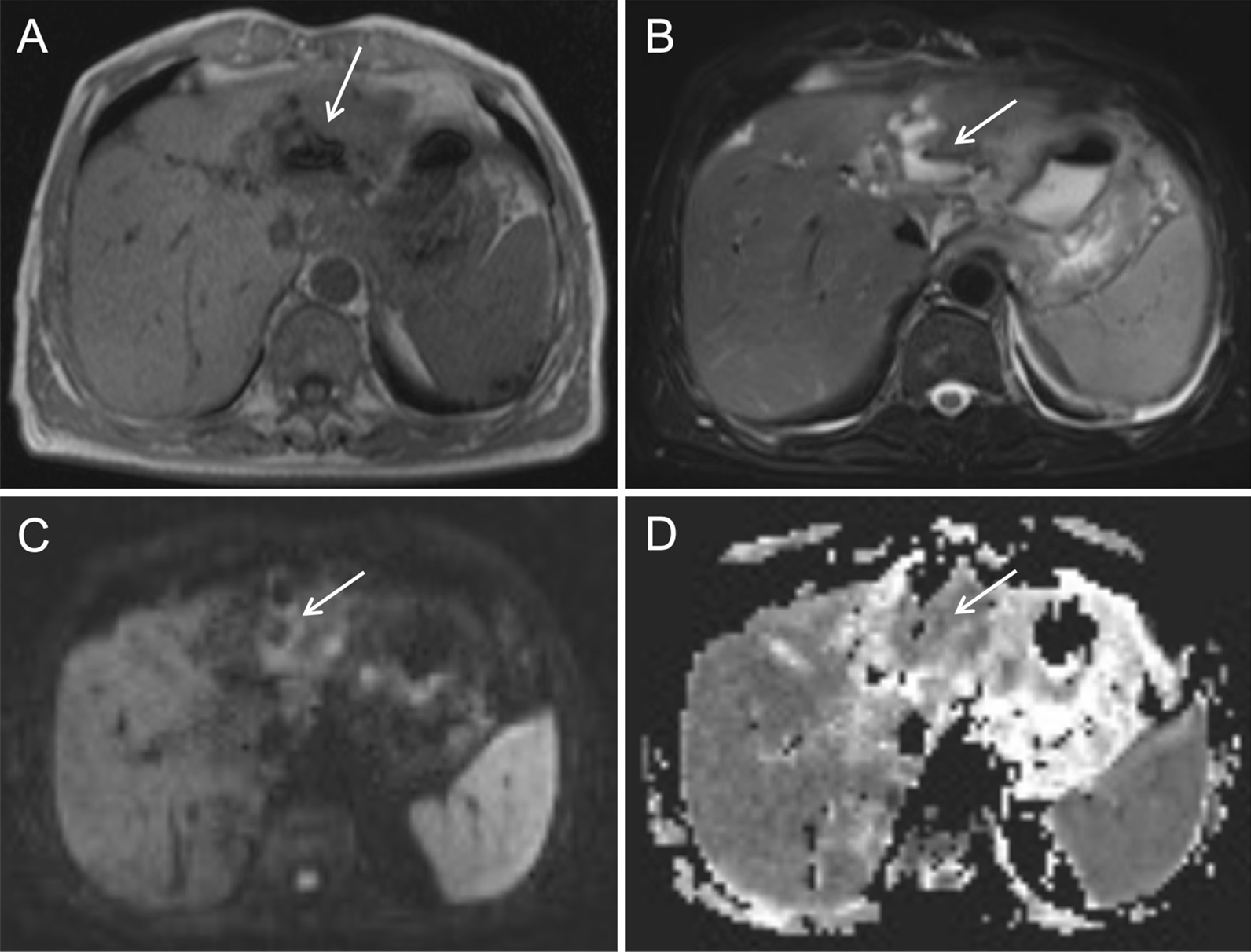
Plain MRI and diffusion-weighted images of case 3

**Fig. 11:**
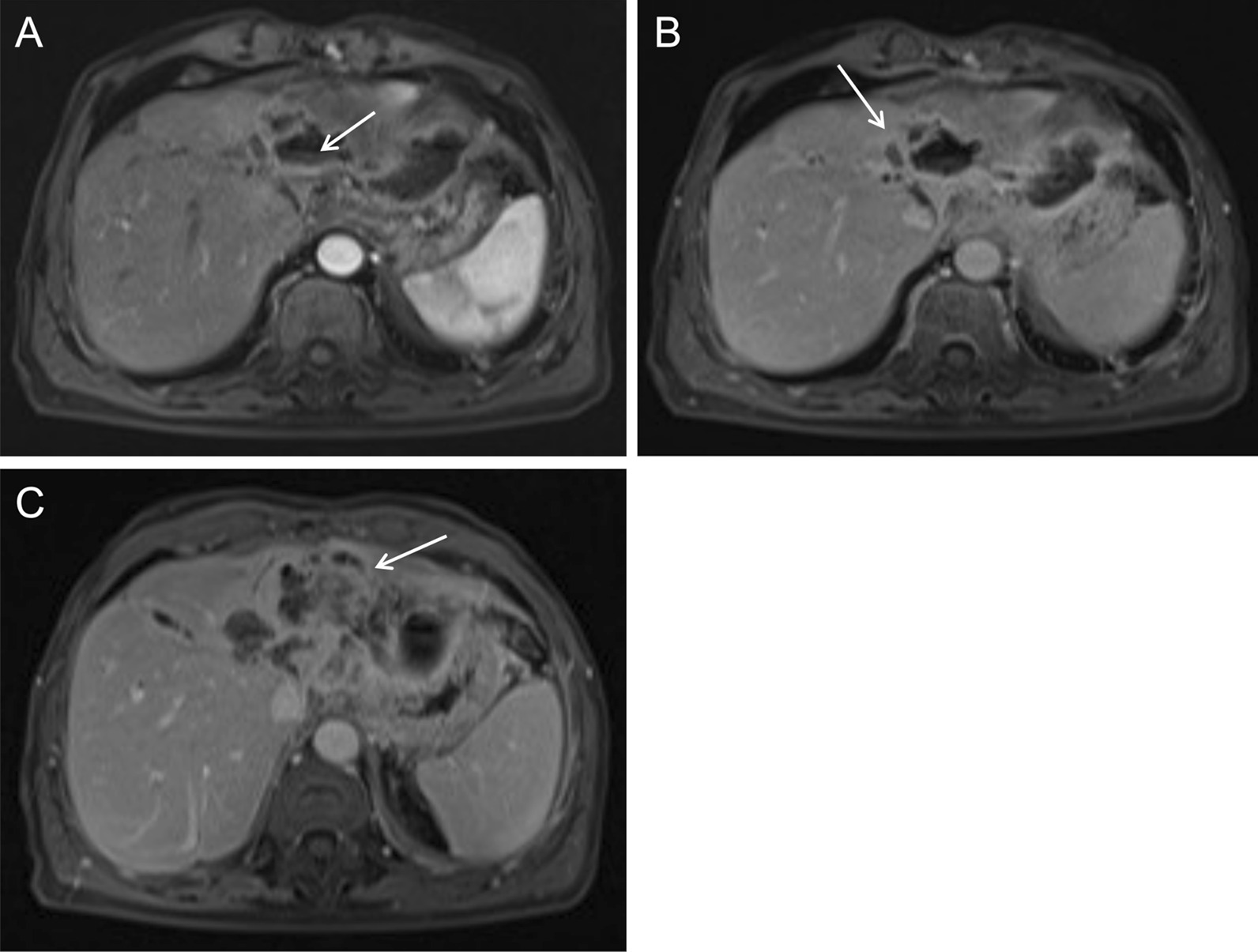
Enhanced MRI images of case 3

T1-weighted images (Fig. 10A, arrow) and fat-suppressed T2-weighted images (Fig. 10B, arrow) showed uneven mixed signal of the mass, with an air-fluid level and adjacent bile duct dilatation. Diffusion-weighted images and apparent diffusion coefficient images showed irregular patchy restricted diffusion of the marginal parenchyma. Hyperintensity was found in the diffusion-weighted images (Fig. 10C, arrow) and hypointensity in the apparent diffusion coefficient images (Fig. 10D, arrow). Dynamic contrast-enhanced MRI showed no enhancement in the necrotic/liquidized area of the lesion (Fig. 11A, arrow) and delayed enhancement in the peripheral area of the necrosis (Fig. 11B, arrow). Irregular, patchy, delayed enhancement with unclear border was found adjacent to the dilated bile duct (Fig. 11C, arrow).

### Imaging features

The most important imaging features of intrahepatic cholangiocarcinoma presenting as pyogenic liver abscess and their frequencies in the 8 patients are summarized in Table 2.

**Table 2:** Imaging features of intrahepatic cholangiocarcinoma presenting as pyogenic liver abscess

Imaging modality	Imaging features	Frequency
Ultrasonography	A prominent edema belt surrounding the mass and an intralesional irregular necrotic/liquidized area	2/3
	Low blood supply to the mass parenchyma	2/3
	Multiple lymph nodes enlargement in the hepatic hilar area and the retroperitoneal space	3/3
CT/MRI	Large, patchy necrotic area without enhancement in the mass; no double target sign of liver abscess; rough and irregular inner wall; multiple dot/patchy and wall nodule enhancements in the mass	8/8
	The abscess wall and the marginal parenchyma were supplied by the hepatic artery; the intralesional arteries were rough and disrupted; a rapid-increase-rapid-decrease pattern of the peripheral lesion enhancement	4/8
	Bile duct dilatation adjacent to the mass; intrahepatic cholangiolithiasis in two patients showed CT hypodensity, T1 hypointensity, T2 hyperintensity, and delayed enhancement	4/8
Diffusion-weighted imaging	Significant restricted diffusion in the necrotic area of the mass; irregular ring-like restricted diffusion in the marginal parenchyma; hyperintensity in the diffusion-weighted images and hypointensity in the apparent diffusion coefficient images	7/7
T1-weighted imaging	Multiple patchy hemorrhagic foci in the mass	3/7
Ultrasonography/CT/MRI	Multiple lymph nodes enlargement in the hepatic hilar area and the retroperitoneal space	5/8

## Discussion

Intrahepatic cholangiocarcinoma accounts for 5–15% of and ranks the second in primary liver malignancies [10]. It is more often seen in the 40 s to the 70 s with a female predominance. Patients with intrahepatic cholangiocarcinoma are usually negative for chronic hepatitis, liver cirrhosis, and alpha-fetoprotein. Our patients were also in this age range and only one patient had cirrhosis and hepatitis B, which is consistent with population survey results in China [11]. However, intrahepatic cholangiocarcinoma is closely associated with hepatitis B virus infection and cirrhosis in Western populations [12]. Intrahepatic cholangiocarcinoma is highly malignant and eventually causes wasting away and compromised immunity, leading to increased risks of infection and abscess in the tumor. Inconsistent with the aforementioned female predominance in intrahepatic cholangiocarcinoma, six out of our eight patients were males. Notably, six patients had type 2 diabetes and five of them were males. Older men have much higher prevalence of diabetes than women [13] and thus are at higher risk of compromised immunity and liver abscess.

Due to the distinctive prognosis of and treatment for intrahepatic cholangiocarcinoma and liver abscess, differential diagnosis of these two diseases is of crucial importance for patient outcomes. Intrahepatic cholangiocarcinoma complicated by liver abscess is uncommon, which usually are separate lesions. Abscess inside an intrahepatic cholangiocarcinoma lesion is rare. It is not difficult to different separate lesions of intrahepatic cholangiocarcinoma and liver abscess because they have distinctive radiological features. However, intrahepatic cholangiocarcinoma with an inside abscess can be easily missed due to the more noticeable abscess in imaging studies. In addition, the infection symptoms of abscess may cover that of intrahepatic cholangiocarcinoma. All the eight patients in our study were initially diagnosed with liver abscess and were treated conservatively. They were later surgically managed due to the poor response to previous treatment and then were pathologically diagnosed with intrahepatic cholangiocarcinoma. Summarizing the imaging features of intrahepatic cholangiocarcinoma presenting as pyogenic liver abscess is useful to improve the diagnosis and patient outcomes.

Ultrasonography found a prominent edema belt surrounding the lesion, an intralesional irregular necrotic and liquidized area, and a posterior hyperechoic belt in two out of three patients who were examined by ultrasound. These findings are typical of liver abscess and have only limited value in diagnosing intrahepatic cholangiocarcinoma presenting as liver abscess. However, all these three patients were found with multiple lymph nodes enlargement in the hepatic hilar area and the retroperitoneal space by ultrasound. This indicates the possibility of liver malignancy besides the abscess, which requires further imaging studies and follow up.

In CT/MR images, a typical liver abscess shows a ring-shaped wall with delayed enhancement, smooth inner wall, and a low-density outer ring caused by edema. In contrast, our patients with intrahepatic cholangiocarcinoma presenting as liver abscess did not show the typical double target sign of liver abscess, and the inner wall of the abscess was quite rough and irregular. In addition, our patients had multiple dot/patchy and wall nodule enhancements were in the lesion, which is different from the beehive enhancement of pure liver abscess. The radiological findings of intrahepatic cholangiocarcinoma presenting as liver abscess may be related to cancer cell infiltration. The subtle differences in the imaging features of intrahepatic cholangiocarcinoma presenting as liver abscess and pure liver abscess are critical to make the right diagnosis.

In four of our patients, the hepatic parenchyma around the necrotic area was supplied by the hepatic artery. The arterial phase images showed dilated tortuous hepatic artery and rough disrupted intratumoral vessels. Irregular ring enhancement was found in the hepatic parenchyma around the necrotic area. Multi-phase enhanced scan showed a rapid-increase-and-rapid-decrease pattern. On the contrary, the blood supply to a liver abscess is more regular and the abscess wall shows delayed enhancement. It could be that the tumor increases blood supply of the hepatic. There are more cancer cells and less fibrotic tissue in the surrounding hepatic parenchyma. Another consideration is compensational increase in the hepatic artery blood flow, which is caused by the tumor or the adjacent dilated bile duct compressing the portal vein. Four of our patients had this radiological sign. Intrahepatic cholangiocarcinoma typically has less blood supply and is rich in fibrotic tissue. In addition, cholangiocarcinoma has nonsignificant early enhancement or mild to moderate enhancement in the marginal part and shows a trend of delayed enhancement [14, 15].

In the diffusion-weighted images, the marginal parenchyma of the necrotic area showed high signal of restricted diffusion. This is because of the tightly packed cancer cells and reduced extracellular space, and thus restricted movement of water molecules. Seven patients in our study had the characteristic imaging features of irregular patchy restricted diffusion in the marginal parenchyma of the necrotic area in addition to the prominent restricted diffusion in the necrotic area, showing high signal in the diffusion-weighted images and low signal in the apparent diffusion coefficient images. These imaging features are distinctive from that of abscess wall and have important values in differential diagnosis.

Bile duct dilatation was seen in four patients in our study, two of which also had cholangiolithiasis near the mass. Typical radiological features of cholangiocarcinoma were seen, including patchy slight CT hypodensity, slight T1 hypointensity, slight T2 hyperintensity, and patchy delayed enhancement. Cholangiocarcinoma arises from the bile duct epithelium and can cause stenosis and occlusion. Cholangiolithiasis is rarely seen with liver abscess. These imaging features are useful in differential diagnosis of cholangiocarcinoma.

Multiple patchy hemorrhages were seen in three patients in our study, showing patchy slight T1 hyperintensity without enhancement. Multiple lymph nodes enlargement in the hepatic hilar area and the retroperitoneal space were seen in five patients. These two signs are not common in patients with liver abscess and may provide some clue for diagnosing cholangiocarcinoma.

## Conclusion

Intrahepatic cholangiocarcinoma mimicking a liver abscess is rare. Patients often have fever and leukocytosis, and lobar atrophy of the liver is uncommon. The signs and symptoms of liver abscess covers that of intrahepatic cholangiocarcinoma and may lead to misdiagnosis. Careful examination and differentiation of the imaging features of intrahepatic cholangiocarcinoma and liver abscess are essential for the correct diagnosis.

## Data Availability

The data of this study is available from the corresponding author.

## Abbreviations

CT: Computed tomography
MRI: Magnetic resonance imaging
